# Unplanned Operative Delivery is Associated with Decreased Perception of Control over Labor

**DOI:** 10.21203/rs.3.rs-2849715/v1

**Published:** 2023-05-09

**Authors:** Anna R. Whelan, Olivia Recabo, Nina K. Ayala, Melissa A. Clark, Adam K Lewkowitz

**Affiliations:** Women & Infants Hospital of Rhode Island, Alpert Medical School of Brown University; New York Medical College; Women & Infants Hospital of Rhode Island, Alpert Medical School of Brown University; Women & Infants Hospital of Rhode Island, Alpert Medical School of Brown University; Women & Infants Hospital of Rhode Island, Alpert Medical School of Brown University

**Keywords:** cesarean delivery, operative vaginal delivery, labor agentry, postpartum depression

## Abstract

**Background:**

Unplanned operative delivery is associated with postpartum depression (PPD), but the mechanism is unknown. We aimed to assess the sense of control over labor for those who had unplanned delivery (unplanned cesarean or operative vaginal delivery: uCD/OVD) versus spontaneous vaginal delivery (SVD).

**Methods:**

Secondary analysis of a cross-sectional survey study of term patients admitted for delivery at a tertiary center. After delivery, patients completed the Labour Agentry Scale (LAS), a validated tool to assess perceived control over labor and birth. Demographics, obstetric and neonatal outcomes and LAS scores were compared between patients who underwent uCD/OVD versus SVD. Multivariable logistic regression to assess the relationship between uCD/OVD and LAS score controlling for confounders that differed in the bivariate analysis.

**Results:**

Of the 149 patients, 50 (33.6%) underwent uCD/OVD. There were no differences in maternal age, race/ethnicity, insurance status or education level between those who had uCD/OVD versus SVD. Patients who had uCD/OVD had higher median body mass index (BMI) than those who had SVD (33.2 vs 30.1 kg/m^2^, p = 0.03). There were no differences in rate of medical or psychiatric morbidity between groups. Additionally, there were no differences in reason for admission, however those who had uCD/OVD had significantly longer times to delivery than those who underwent SVD (22 vs 14 hrs, p < 0.01). Gestational age at delivery was also significantly higher for those who underwent uCD/OVD compared to SVD (40.2 vs 39.6 wks, p = 0.02). For the primary outcome, LAS scores were lower for those who underwent uCD/OVD compared to SVD (146 vs. 164, p < 0.01). This remained significant even after controlling for length of labor, BMI and gestational age at delivery (p < 0.01).

**Conclusions:**

Even after accounting for length of labor, uCD/OVD is associated with a reduction in perceived control over labor, which may mediate the known increased risk of PPD. Further qualitative research is needed to examine how to better support patients’ wellbeing after uCD/OVD.

## Background

There is increasing understanding that patient’s perceived control over their labor and delivery may mitigate postpartum mental health disorders (PPMD)^1^. Compared to those who have spontaneous vaginal deliveries (SVDs), patients who undergo unplanned cesareans or operative vaginal delivery (uCD/OVD) are at increased risk of PPMD.^2^ Though the difference in risk of PPMD based on delivery mode has been attributed to perceived control over childbirth, data supporting the potential association between uCD/OVD and perceived control over childbirths remains limited^3^. Using the Labour Agentry Scale (LAS)-a validated instrument that assesses patient perception of control during childbirth^4^–we aimed to examine whether patients who underwent uCD/OVD perceived less control during childbirth compared to patients who underwent SVD.

## Methods

This was a preplanned secondary analysis of a cross-sectional survey study of patients admitted to the labor and delivery unit at Women & Infants Hospital of Rhode Island (WIH) from June through July 2021. WIH is a large academic center with a catchment area that includes all of Rhode Island as well as Southern Massachusetts and Connecticut and has an annual delivery rate of approximately 8,500 deliveries. Prior to enrollment initiation, the study was approved by the institutional review board (#1691795).

Eligibility was assessed via chart review and participants were approached on the postpartum unit. Eligible participants were nulliparous, English-speaking, and had singleton pregnancies at gestational age ≥ 37 weeks and were approached on the postpartum unit. The primary outcome was the total score for the LAS. All After obtaining consent, participants filled out a detailed survey of past medical and psychiatric history and the Labour Agentry Scale (LAS), a validated 29-item instrument that assesses childbirth control^7^. Trained medical personnel then performed a detailed chart review of their obstetric course and collected information on labor interventions, analgesia, mode of delivery, postpartum complications (hemorrhage, preeclampsia, infection) as well as neonatal outcomes including NICU admission, need neonatal treatment.

Participants who underwent uCD/OVD were compared to those who underwent SVD using Fisher’s exact and Wilcoxon Rank-sum tests. Multivariable linear regression was performed to assess for confounders identified from the bivariate analysis.

## Results

**Figure 1:**
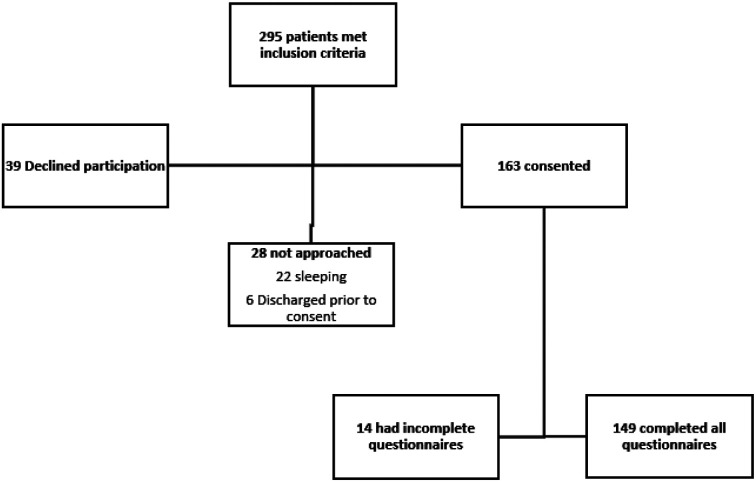
Flowsheet of subject enrollment.

From the 149 participants included in this secondary analysis, 50 (33.6%) underwent uCD/OVD (Fig. 1). Maternal BMI was significantly higher in the group which underwent uCD/OVD as compared to SVD (median 33.2 vs 30.1, respectively, p < 0.03). There were no differences between groups in terms of maternal age, race, insurance payer or maternal education (Table 1). Additionally, rates of medical comorbidities (which included chronic hypertension, gestational hypertension and preeclampsia, pregestational and gestational diabetes, thyroid disease and SARS-CoV-2 infection) and rates of depression and/or anxiety did not differ between groups (Table 2).

There were also no differences in the distribution of admission types between groups (admitted for labor, planned induction or induction admitted from triage). However, length of labor was significantly higher among those who underwent uCD/OVD as compared to SVD (median 22 hours vs 14 hours, p < 0.02). Gestational age at delivery was also higher among those who underwent uCD/OVD (median 40.2 weeks) compared to SVD (median 39.6 weeks, p < 0.02). There were no differences in rates of NICU admission or the need for neonatal therapy (which included the need for supplemental oxygen, phototherapy and neonatal antibiotics).

Scores on the LAS were significantly lower for participants who underwent uCD/OVD (median 146) than those who underwent SVD (median 164, p < 0.01). These findings remained significant after controlling for BMI, length of labor, and GA (Scores were 16.09 (± 4.64) points lower among those who underwent uCD/OVD compared to SVD, p < 0.01) (Table 2).

## Discussion

In this study, those who underwent uCD/OVD had significantly lower scores on the LAS than those who underwent SVD. This finding remained significant even after controlling for maternal BMI, gestational age at delivery and length of labor. This indicates that participants who underwent uCD/OVD experienced less control over their labor process.

These findings are consistent with a prior study by Floris et al^3^, though their cohort was smaller – with 78 participants – and their analyses did not control for factors such as length of labor that may be associated with labor experience ^3^.

Mode of delivery has been demonstrated to be a key mediator of development of PPMD,^5^ impacting up to 15–20% of birthing people^6^. If the perception of losing control over childbirth mediates development of PPMD is correct, interventions to increase the experience of control must be examined. These could incorporate psychotherapeutic approaches such as cognitive behavioral therapy or education-based interventions designed to increase patient engagement in decision-making during labor. Regardless of the exact intervention, it is crucial to further explore whether improving patient perceptions of control during childbirth may decrease rates of PPMD.

Strengths of this study include that each participant had a detailed health history and labor interventions/outcomes collected from the medical chart by trained researchers. This allowed for us to compare between groups and account for differences between groups in our analysis.

For this study, we combined operative vaginal delivery and cesarean delivery as we were limited in the number of operative vaginal deliveries in our sample and both types of deliveries are high intervention births. However, there are likely differences in how these two groups experience their labor. Future studies with larger samples of operative vaginal deliveries should be performed to further assess these findings. A second limitation is that how LAS was only collected at a single time point during a participant’s postpartum hospitalization. How a participant views their experience of childbirth may change over time. Lastly, we did not collect data on depressive symptoms so cannot correlate our findings with rates of postpartum depression and anxiety.

## Conclusion

Even after controlling for confounding factors, participant experience of control over labor was lower among those who underwent unplanned cesarean or operative vaginal delivery compared to those who underwent spontaneous vaginafl delivery. Increasing patient experience of control, particularly among those who have unplanned operative deliveries, may represent a target for interventions to decrease rates of postpartum mental health disorders.

## Figures and Tables

**Table 1.** Demographics and Delivery Characteristics

	Cesarean Delivery/ Operative Vaginal Delivery (n=50)	Spontaneous vaginal delivery (n=98)	p- value
Maternal age, median (IQR)	29.5 (26,33)	28.5 (24,31)	0.08
Maternal BMI, median (IQR)	33.2 (29.1,40.9)	30.1 (27.5,35.7)	**0.03**
Maternal race/ethnicity			
Black	2 (4.0)	6 (6.1)	0.72
Latina	12 (24.0)	14 (14.3)	0.17
Indigenous	1 (2.0)	4 (4.1)	0.66
Asian/Pacific Islander	2 (4.0)	0	-
Caucasian	33 (66.0)	74 (75.5)	0.25
Primary insurance			0.64
Public	13 (26.0)	32 (32.7)	
Private	37 (74.0)	65 (66.3)	
Self-pay/none	0	1 (1)	
Highest level of education			0.35
12^th^ grade or less	19 (38.0)	29 (29.6)	
Greater than 12^th^ grade	31 (62.0)	69 (70.4)	
Medical comorbidity*	20 (40.0)	28 (28.6)	0.19
Depression and/or anxiety	29 (58.0)	44 (44.9)	0.17
Delivery characteristics			
Admitted for:			0.14
Labor	23 (46.0)	59 (60.2)	
IOL (sched)	21 (42.0)	25 (25.5)	
IOL (from triage)	6 (12.0)	14 (14.3)	
Length of labor (hours), Median (IQR)			**<0.01**
	22 (15,34)	14 (10,22)	
Gestational age at delivery, Median (IQR)	40.2 (39.3,41)	39.6 (38.7,40.6)	**0.02**
NICU admission	7 (14.6)	6 (6.1)	0.12
Neonatal therapy**	6 (12.0)	14 (14.3)	0.80

Data are N(%) unless otherwise stated. Significance at p<0.05.

Fisher’s exact and Wilcoxon Ranksum tests used for analysis.

IQR = interquartile range, BMI = body mass index, IOL = induction of labor, NICU = neonatal intensive care unit

* Maternal medical comorbidities include chronic hypertension, gestational hypertension, preeclampsia, pregestational diabetes and gestational diabetes, thyroid disease and SARS-CoV-2 infection.

** Neonatal therapy includes the need for supplemental O2, phototherapy for jaundice, neonatal antibiotics

**Table 2:** Labour Agentry Scale Scores by Mode of Delivery and Multivariable Linear Regression of Labour Agentry Scale Scores

	Cesarean Delivery/ Operative Vaginal Delivery (n = 50)		Spontaneous vaginal delivery (n = 98)	p- value
Total LAS	146 (131,161)		164 (146,181)	**<0.01**
Median (IQR)				
Multivariable Linear Regression				
	Beta	SE	t	p
Constant	123.84	72.17		
CD or OVD	−16.09	4.64	−3.47	**<0.01**
Duration of labor (hrs)	−0.23	0.12	−1.92	0.06
Maternal BMI	−0.21	0.29	−0.74	0.46
Gestational age at delivery	1.24	1.79	0.69	0.49

Significance at p < 0.05.

Wilcoxon Ranksum test used for analysis of LAS score between participants who underwent CD/OVD as compared to SVD
